# Rapid Resistance Detection of *Amaranthus retroflexus* to Fomesafen via Kompetitive Allele-Specific PCR (KASP)

**DOI:** 10.3390/plants14040515

**Published:** 2025-02-08

**Authors:** Zhanzhan Zhang, Yaxin Ban, Jianguo Wei, Qun Wu, Liyao Dong, Zhike Feng

**Affiliations:** Key Laboratory of Soybean Disease and Pest Control (Ministry of Agriculture and Rural Affairs), College of Plant Protection, Nanjing Agricultural University, Nanjing 210095, China

**Keywords:** *Amaranthus retroflexus*, fomesafen, target-site resistance, KASP

## Abstract

*Amaranthus retroflexus* is a highly invasive annual broadleaf weed in soybean fields, significantly reducing soybean yield and quality. Diphenyl ether herbicides, particularly fomesafen, are extensively applied to control *A. retroflexus*. Fomesafen resistance of *A. retroflexus* is emerging in Northeast China, and rapid resistance detection is urgent for managing these resistant weeds. An Arg-128-Gly mutation in the *ppo2* gene of *A. retroflexus* has been shown to confer fomesafen resistance. In current study, we developed a rapid detection method based on Kompetitive Allele-Specific PCR (KASP) technology to detect the Arg-128-Gly mutation in the *ppo2* gene of *A. retroflexus*. Initially, we developed this KASP detection method using cDNA as the template; however, the entire process requires significant costs and considerable operational time. To mitigate these expenses and simplify the workflow, we subsequently optimized this KASP rapid detection method by utilizing genomic DNA as the template. This new resistance detection technique directly utilizes *A. retroflexus* genomic DNA as the template, and, by adding specific labelled primers, probes, and enzymes, it can determine whether the *ppo2* gene harbors an Arg-128-Gly mutation, thereby rapidly identifying fomesafen resistance in *A. retroflexus*. Furthermore, we compared the detection efficiency of the new KASP assay, whole plant dose–response assay, and DNA sequencing, all of which produced consistent outcomes, supporting the accuracy and reliability of the KASP rapid detection method. Collectively, we established a rapid resistance detection method based on KASP technology, which is of high reliability and time-saving, and will significantly advance precise management of resistant weeds.

## 1. Introduction

Soybean, rich in high-quality protein and used for vegetable oil, is a key agricultural product with steadily increasing demand [1]. Average soybean yields in China are approximately 1950 kg/ha, roughly half that of the US and Brazil [2].

Weed infestation can cause crop reductions of over 10%, reaching up to 50% in severe cases, and, in extreme situations, can result in total production loss [1]. There are about 70 weed species in soybean fields, including annual dicotyledonous, broadleaf weeds, and perennial weeds, including annual grasses such as *Echinochloa crus-galli* and *Digitaria sanguinalis*; annual dicotyledonous such as *Chenopodium album*, *Amaranthus retroflexus*, and *Commelina communis*; and perennial weeds such as *Phragmites australis* and *Cirsium setosum* [3]. Weed species common in soybean fields exhibit characteristics such as prolonged emergence period [1], vigorous growth during high-temperature rainy seasons, and notably high density.

*Amaranthus retroflexus* is an annual broadleaf weed with strong adaptability [4], originating in North America and now widely distributed in China [5]. It spreads through crop seeds, water, wind, organic fertilizers, livestock manure, and birds. This weed competes with crops for light, water, and nutrients, affecting crop growth [6]. Common control methods include chemical agents like fomesafen and thifensulfuron-methyl [7]. Fomesafen, a diphenyl ether herbicide developed by ICI Plant Protection in the UK, was first registered and used in China in 1998 to control broadleaf weeds in dry field crops like peanuts, corn, and soybeans, and also inhibits some grass weeds [8]. The herbicidal target of fomesafen is protoporphyrinogen IX oxidase (PPO), a key enzyme in the tetrapyrrole biosynthesis pathway, which is crucial for chlorophyll and heme production. PPO belongs to a highly conserved family of membrane-bound enzymes [9,10]. There are two subtypes, PPO1 and PPO2. PPO1 is encoded by *ppo1* and located on the chloroplast membrane. PPO2 is encoded by *ppo2* and located on the mitochondrial membrane [11]. In some prokaryotes and plants, *ppo2* can target both chloroplasts and mitochondria. When PPO inhibitors are applied, herbicides compete with protoporphyrinogen IX for the PPO active site, inhibiting PPO activity. This causes rapid accumulation of protoporphyrinogen IX in plastids and mitochondria, leading to its leakage into the cytoplasm. Protoporphyrinogen IX, which is photo-insensitive, is oxidized in the cytoplasm via a non-enzymatic pathway to form photosensitive protoporphyrin IX. Under aerobic and light conditions, this accumulation near the cell membrane produces singlet oxygen and other toxic oxygen species, damaging the cell membrane, causing cellular contents to leak, and ultimately inducing cell death [12]. PPO inhibitors represented by diphenyl ethers have a broad herbicidal spectrum, high efficacy, fast action rate, low toxicity, and short soil residual period. In addition to diphenyl ether herbicides [13], PPO inhibitors commonly used in China also include N-phenyl-oxadiazolones, N-phenyl-imides, N-phenyl-triazolinones herbicides, etc. [14].

With the extensive and continuous usage of herbicides, weeds experience constant selective pressure, evolving resistance to herbicides. Weed resistance mechanisms are categorized into target-site resistance and non-target site resistance. Target-site resistance involves gene mutations that alter enzyme structures, preventing herbicide binding as well as overexpression of target enzyme genes producing excess enzymes that exceed herbicide binding capacity [15]. Non-target site resistance encompasses all resistance mechanisms that do not involve alterations at the target site. To date, 15 weed species (11 dicotyledonous and 4 monocotyledonous) exhibiting resistance to PPO inhibitors have been documented globally [16]. The initial observation of reduced efficacy of PPO inhibitor herbicides was reported in soybean fields in the United States: *Amaranthus rudis* developed resistance to acifluorfen sodium and exhibited 82-, 8-, and 4-fold resistance to lactofen, fomesafen, and sulfentrazone, respectively [17]. In 2011, it was first reported in China that *Acalypha australis* exhibited resistance to lactofen and fluoroglycofen [18]. In 2016, the resistance of *A. retroflexus* to fomesafen was first documented [19]. A range of target-site mutations have been identified that confer resistance to PPO inhibitors in weeds, including the loss of the Gly-210 amino acid residue in PPO2 [20] and the substitution of the Arg-128-Gly/Met [21,22,23,24], Gly-399-Ala [25], and Val-361-Ala [26] amino acids in PPO2, and the substitution of Ala-212-Thr in PPO1 [27].

Currently, whole plant dose–response assays are widely employed to assess weed resistance level. This method is among the primary techniques recommended by the Herbicide Resistance Action Committee (HRAC) for evaluating the herbicide resistance of weeds [28]. Initially, weed seeds are collected from suspected herbicide-resistant populations or from fields that have never been exposed to herbicides, then potted in a controlled greenhouse environment. Routine application treatments are administered either pre-emergence or post-emergence by spraying different doses of the herbicide [29]. This method is unable to identify the causes and mechanisms of resistance, and it requires a significant amount of time, often extending until the next season to define the resistance status, which results in missing the optimal timing for resistant weed management. Consequently, there is an urgent need for a rapid method that can assess herbicide resistance to effectively manage resistant weeds and minimize crop reduction.

Kompetitive Allele-Specific PCR (KASP) genotyping technology is a fluorescence-based method that facilitates high-precision biallelic genotyping of SNP and insertion/deletion variants in diverse genomic nucleic acid samples through competitive allele-specific amplification [30,31,32]. The technique offers simple operation, stable and accurate analysis, and low cost, and supports high-throughput automated processing. Generally, a single KASP program only needs 1.5 h. KASP was primarily applied to SNP and InDel genotyping research, but it has now been introduced as a principal tool for molecular-assisted breeding, fine mapping of trait genes, and identification of germplasm resources [33]. During PCR amplification, the binding efficiency of primers to the template strand is significantly higher when the last base of the primer matches that of the template strand compared to when it does not. Additionally, different specific fluorescent gene sequences are introduced at the 5′ end of both forward primers with different terminal bases. Consequently, during PCR amplification, primers whose terminal bases match those of the template strand will bind more effectively, resulting in stronger specific fluorescence signal. This allows for accurate determination of the terminal base of the template strand.

**Figure 1:**
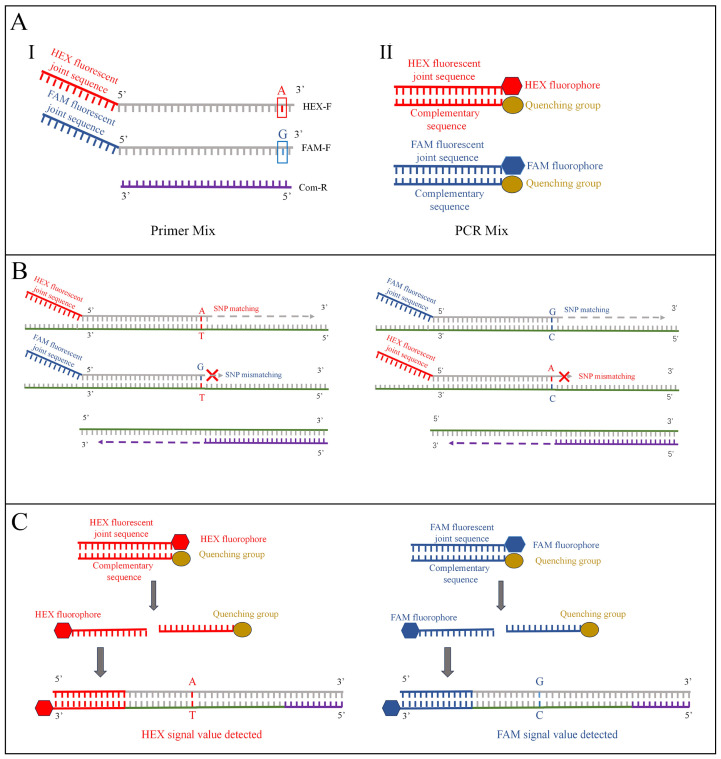
**Workflow of KASP genotyping technology-based herbicide resistance detection.** (**A**) (I) A Primer Mix consisting of two forward primers (HEX-F and FAM-F) with distinct terminal bases and one common reverse primer (Com-R). The 5′ ends of the two forward primers are respectively linked to different detection primer sequences. (II) PCR Mix containing two detection probes with distinct fluorescent labels. (**B**) The DNA template anneals with the matching primers from the Primer Mix, and during extension, the detection primer sequences are incorporated into the DNA. (**C**) The DNA strands containing the detection primer sequences specifically hybridize with the fluorescently labeled probes in the PCR Mix, leading to enhanced fluorescence signals after multiple rounds of PCR amplification.

In this study, a KASP-based identification system specifically targeting the Arg-128-Gly amino acid substitution in PPO2 of *A. retroflexus* was developed. This method involved designing a pair of forward primers with distinct fluorescent signal sequences and different terminal bases, along with a conventional reverse primer. The intensity of the different fluorescent signals generated by competitive allele-specific PCR technology reflects the homology of the bases at the mutation site, thereby determining whether the Arg-128-Gly amino acid substitution occurs at this locus (Figure 1). This system is designed to detect whether this specific mutation occurs in *A. retroflexus*, leading to herbicide resistance. By accelerating resistance monitoring, it helps to enable the exact selection of appropriate herbicides at the optimal time to mitigate resistant weeds. This approach is crucial for effective integrated weed management, reducing the infestation caused by noxious resistant weeds to farmlands, and improving crop yield and quality.

## 2. Results

### 2.1. Fomesafen Sensitivity of A. retroflexus Populations

#### 2.1.1. Preliminary Sensitivity Screening

Whole-plant dose–response bioassays were conducted to evaluate the sensitivity of 42 *A. retroflexus* populations to fomesafen. By 2 days post fomesafen application, leaves of *A. retroflexus* populations exposed to high doses exhibited wilting and yellowing, progressively leading to complete withering and death. In contrast, populations treated with low doses developed necrotic spots but recovered within one week. By 14 days, plants treated with water exhibited normal growth; however, as the fomesafen concentration increased, its inhibitory effect on the fresh weight of *A. retroflexus* became progressively more pronounced.

#### 2.1.2. Whole-Plant Dose–Response Assay

Among the suspected resistant populations identified through preliminary sensitivity screening, 14 populations exhibiting robust seed viability were selected for further evaluation. The relative resistance indices of these populations were determined using whole-plant dose–response assays (Table S1). FZ-41 was chosen as the reference sensitive population to evaluate the resistance index of 14 populations suspected to be resistant to fomesafen and in optimal growth condition. Among these populations, four exhibited decreased sensitivity, with GR_50_ values ranging from 9.73 to 11.22 g a.i./ha. Four populations developed low-level resistance, with GR_50_ values between 15.92 and 20.28 g a.i./ha. Another four populations demonstrated high levels of resistance, with GR_50_ values ranging from 110.41 to 125.56 g a.i./ha. FZ-41 remained the most sensitive population, with a GR_50_ of 2.83 g a.i./ha. FZ-11 showed the highest resistance level, with a GR_50_ of 125.56 g a.i./ha and a resistance index of 44.31. Other highly resistant populations included FZ-7, FZ-5, and FZ-8, with GR_50_ values of 115.80, 115.38, and 110.41 g a.i./ha, respectively, and corresponding resistance indices of 40.87, 40.72, and 38.97 (Table S1).

### 2.2. Gene Mutation Analysis of PPO2

**Figure 2:**
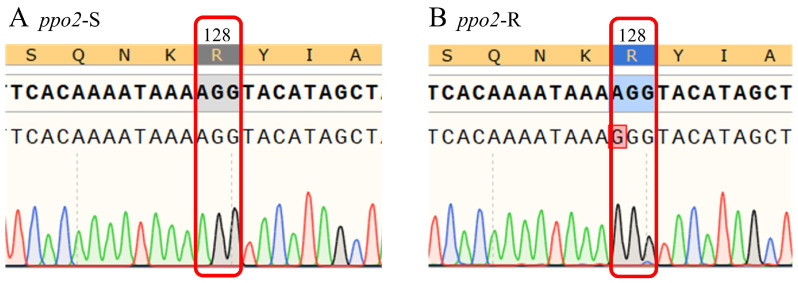
**Amino acid mutation of the *ppo2* gene in resistant populations.** The partial sequence near the 128th amino acid site of the *ppo2* gene in the sensitive population (**A**) and the resistant population (**B**).

After comparing the *ppo2*-S gene from the sensitive population with the *ppo2*-R gene from the resistant population in Nenjiang County, Heilongjiang Province, a single base mutation from A to G was identified in the resistant population (Figure 2). This mutation resulted in the substitution of arginine by glycine at position 128 (Arg-128-Gly). The mutation was consistently observed in all 130 sequenced strains from 13 resistant populations in Nenjiang County, indicating a mutation frequency of 100% (Table S1). Furthermore, the sequencing peak chart revealed a unimodal pattern at this site for each resistant population (Figure 2), suggesting that the mutation was homozygous.

### 2.3. Development of KASP Assay with cDNA as the Template

**Figure 3:**
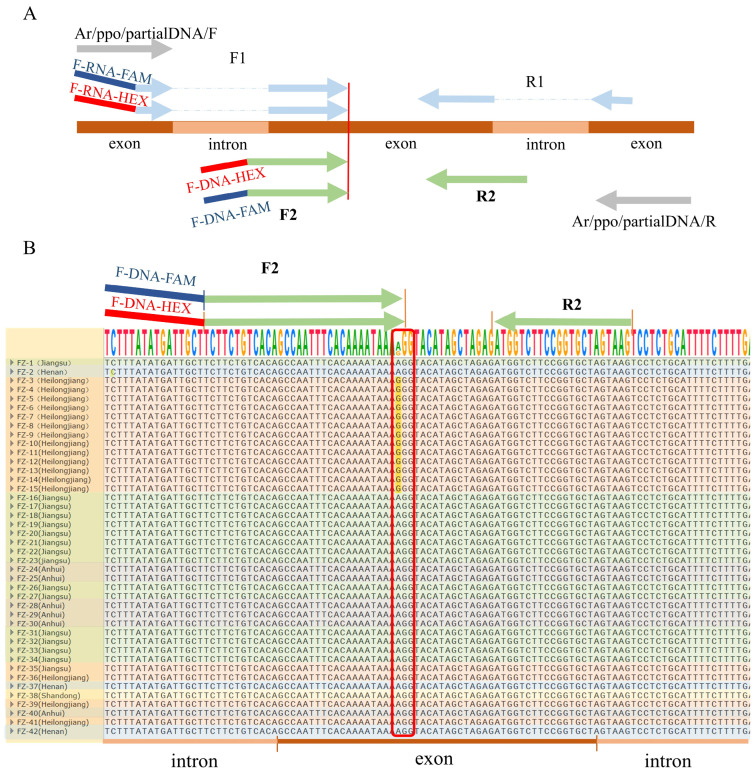
**DNA sequence alignment for KASP primer optimization.** (**A**) Schematic diagram of KASP primer optimization. By comparing the gene sequences of *A. tricolor* on NCBI (NC080059.1, positions 8666371–8678257), the initially designed KASP primers F1 and R1, based on *A. retroflexus* cDNA, were interrupted by introns. To address this issue, primers Ar/ppo/partialDNA/F and Ar/ppo/partialDNA/R were used to amplify a 700 bp fragment of the *ppo2* gene from *A. retroflexus*, which includes site 128. Based on the alignment and consensus of that fragment, new KASP primers F2 and R2 were designed for following assays. (**B**) The partial *ppo2* gene of *A. retroflexus* populations from different provinces is aligned with varying background colors. All resistant population individuals exhibited the same mutations.

**Figure 4:**
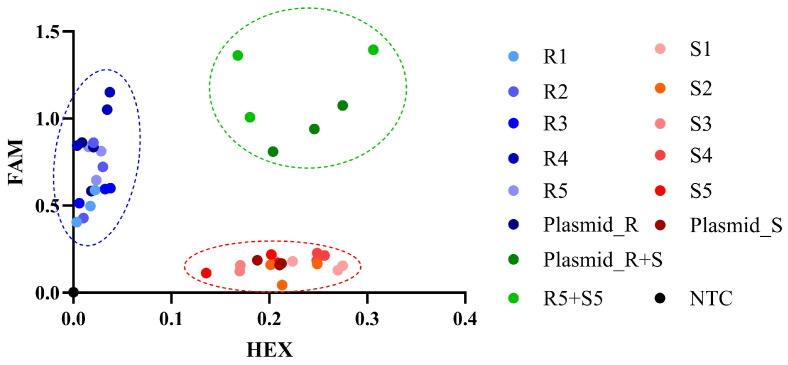
**KASP-based resistance detection using cDNA as a template.** Fluorescence data for FAM and HEX were normalized using ROX values, with ROX serving as the reference dye for scatter plot coordinates. Samples from resistant mutants (R1, R2, R3, R4, R5, Plasmid_R) cluster near the FAM axis, while samples from sensitive unmutated individuals (S1, S2, S3, S4, S5, Plasmid_S) cluster near the HEX axis. Synthetic heterozygous mutant samples (Plasmid_R+S and R5+S5), which are mixtures of resistant mutations and sensitive non-mutated samples, cluster in the center of the plot.

The KASP method was employed to detect each cDNA sample (Figure 1 and Figure 3A). The results in Figure 4 demonstrate that the data points representing samples R1, R2, R3, R4, R5, and Plasmid_R cluster near the FAM axis, whereas those for samples S1, S2, S3, S4, S5, and Plasmid_S cluster near the HEX axis. Samples R1 through R5 and Plasmid_R originate from resistant mutant populations, while samples S1 through S5 and Plasmid_S come from sensitive non-mutated populations. Additionally, the mixed samples Plasmid_R+S and R5+S5 combine Plasmid_S with Plasmid_R, and S5 with R5, respectively. These findings suggest that this technique can effectively differentiate between homozygous mutations at position 128 and heterozygous mutations in *A. retroflexus* cDNA, as well as distinguish them from non-mutated samples (Figure 4).

### 2.4. Identification of Consensus DNA Sequence Within PPO2 Genomic Sequence

Genomic DNA was extracted from individual plant samples from all of our populations of *A. retroflexus.* The long fragment concluding the 128 site was amplified to determine the consensus DNA sequence within PPO2. Notably, a mutation at position 128, where the base changed from AGG to GGG, was identified in plant samples from the resistant population in Nenjiang City, Heilongjiang Province. We designed primers F2 and R2 based on the consensus DNA sequence, which can be used to amplify the DNA fragments from genomic DNA for KASP assay, even the populations from different geographical locations (Figure 3B).

### 2.5. Optimization of KASP Assay by Using Genomic DNA as the Detection Template

**Figure 5:**
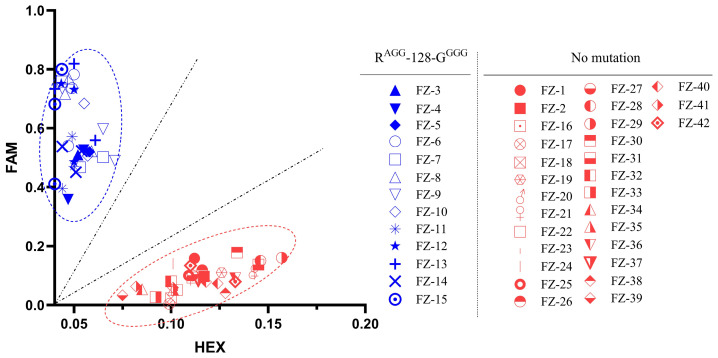
**KASP-based resistance detection using genomic DNA as the template.** The blue cluster represents the single resistant mutant, which is concentrated near the FAM axis. The red cluster represents the single wild type, distributing near the HEX axis. The scatter plots clearly distinguish between resistant mutants and wild type, enabling rapid identification of whether the sample conferred target-site resistance to fomesafen.

PCR was conducted by using genomic DNA from individual plants as the template, with primers F2 and R2. The amplified fragments were utilized for the KASP genotyping assay. Following the reaction, signal intensities were recorded for each sample, The results indicate that DNA samples from mutant resistant *A. retroflexus* collected in Nenjiang City, Heilongjiang Province, clustered closer to the FAM axis, while DNA samples from unmutated strains from other populations clustered closer to the HEX axis (Figure 5). This clustering pattern aligns with our bioassay results and DNA sequencing data (Table S1), which showed that resistant mutant population samples and non-mutant population samples clustered around the FAM and HEX axes, respectively. Therefore, using genomic DNA as the template, the KASP assay can be used to determine whether a plant sample exhibits target-site resistance conferred by an Arg-128-Gly mutation.

## 3. Discussion and Conclusions

Target gene mutations confer herbicide resistance. It was initially identified that the resistance mechanism of *A. tuberculatus* to PPO herbicides is attributed to a nucleotide deletion (ΔG210) in the *ppo2* gene [20]. The substitution of arginine at position 128 of *ppo2* with glycine or methionine (R128G/M) confers resistance to PPO inhibitors in *Amaranthus* weeds [21], and position 128 is homologous to the arginine-98 site of PPO2 in *Ambrosia artemisiifolia* [34]. Substitution with Leucine at this position can also confer resistance to PPO-inhibiting herbicides [35]. All known mechanisms of resistance to PPO inhibitor herbicides have been documented in *A. palmeri* [26]. Considering the dioecious nature, outcrossing capability, and resultant genetic diversity of *Amaranthus* weeds, it is not surprising that these species exhibit multiple target-site mutations and non-target-site resistance mechanisms to the same herbicide family. Any mutation occurring in an individual can be readily spread within and between populations, particularly the Arg-128-Gly mutation, as it requires only a single nucleotide change in the *ppo2* gene [36].

Resistance development of PPO-inhibiting herbicides in China has been relatively slow. Since the initial discovery in 2011 that *Acalypha australis* exhibited resistance to lactofen and fluoroglycofen-ethyl [18], only three weed species have developed resistance to diphenyl ether herbicides: *Descurainia sophia* [37], *A. retroflexus* [22], and *Ipomoea hederacea* [37]. Among the resistant weeds, *A. retroflexus* exhibits the most rapid development of herbicide resistance. Numerous domestic scholars have successively elucidated the target-site resistance mechanism in *Amaranthus*, specifically the occurrence of the Arg-128-Gly mutation in PPO2 [19,38,39,40]. Additionally, non-target-site resistance mechanisms mediated by cytochrome P450, enhanced glutathione S-transferase (GST) activity, and increased herbicide metabolism have also been documented [41,42].

The resistance index of the resistant populations collected in Nenjiang County, Heilongjiang Province, ranged from 40.65 to 101.74 [19]. This finding was consistent with the relative resistance level of the resistant populations in Nenjiang County, Heilongjiang Province (ranging from 38.97 to 44.31). The mutation Arg-128-Gly in the *ppo2* gene of *A. retroflexus*, identified in this study, has been previously reported [22]. Prior research has demonstrated that multiple amino acid substitutions within the five highly conserved regions of PPO confer resistance to PPO-inhibiting herbicides in weeds, including the deletion of the Gly-210 amino acid in PPO2 [19,20], amino acid substitutions of Arg-128-Gly/Met [21,22,23,24], Gly-399-Ala [42] and Val-361-Ala in PPO2 [25], and substitution of Ala-212-Thr in PPO1 [26]. In this study, a mutation at position 128 in the highly conserved region of PPO, changing from arginine (Arg) to glycine (Gly), was identified. Besides *A. retroflexus*, the Arg-128-Gly mutation has been documented in *Ambrosia artemisiifolia* [35] and *A. palmeri* [21]. This mutation has been confirmed to confer resistance to PPO inhibitor herbicides at the whole-plant, molecular, and crystal-structure levels [21].

In the current study, we established a rapid detection method based on KASP technology to evaluate the occurrence of the Arg-128-Gly mutation in the *ppo2* gene of *A. retroflexus*, which confers resistance to diphenyl ether herbicides. The Herbicide Resistance Action Committee (HRAC) recommends whole-plant dose–response assay as one of the primary methods for weed resistance testing [43]. This method simulates field growth conditions, ensuring that resistance detection closely mirrors real-world scenarios, making it a widely adopted approach for assessing weed resistance. Although highly feasible and reproducible, this method requires 3 to 4 months from seed collection to the identification of resistance outcomes. Consequently, it demands considerable time and resources, including significant human and material inputs for plant cultivation, rendering it unsuitable for large-scale resistance screening [29]. The KASP technology employed in this study enables direct field sampling. After DNA extraction, a single specialized KASP assay, which takes approximately one hour to complete, can accurately determine whether the sample contains the mutation. This new method enables the simultaneous detection of a large number of samples, with the entire process from DNA extraction to result acquisition taking only 3–4 h. As a high-throughput detection technology, KASP can process a flexible range of sample sizes, from single samples to thousands, demonstrating remarkable adaptability [30]. Furthermore, by comparing the experimental results with those obtained from whole-plant dose–response assay and DNA sequencing, the consistency across these three methods confirms the reliability of this new approach. Every method has its limitations, and the KASP technology we studied is no exception: we can use this method to detect the occurrence of resistance caused by the Arg-128-Gly type of mutation, but it is unable to detect mutations at other sites and resistance conferred by non-targets. However, this new method can be extended to different types of mutation at different loci of different genes in different weeds by designing specific primers based on different sequence information. This new rapid method for monitoring herbicide resistance will significantly facilitate precise resistant weed management and mitigate weed infestation.

## 4. Materials and Methods

### 4.1. Materials and Instruments

Seeds of *A. retroflexus* were collected from the provinces of Heilongjiang, Jiangsu, Henan, and Anhui. All seeds were manually harvested, air-dried in the shade, and stored in paper bags at 4 °C until use. Detailed population information is provided in Table S1. For the purposes of the study, we acquired 250 g/L fomesafen aqueous solution supplied by Hengda, China, with a recommended application rate of 562.5 g a.i./ha.

The 3WP-2000 Walking Spray Tower, developed by the Nanjing Institute of Agricultural Mechanization under the Ministry of Agriculture, was used. The spindle speed was 96 mm/r, the spray height was 300 mm, the effective spray width of the nozzle was 350 mm, the flow rate of the nozzle was 390 mL/min, and the traveling distance was 1340 mm. The walking speed of stem and leaf treatment was 291 mm/s.

### 4.2. Sensitivity to Fomesafen

#### 4.2.1. Preliminary Sensitivity Test

The sand, clay soil, and organic matter were thoroughly mixed at a ratio of 1:1:2 (pH 6.3, organic matter content 1.1%) and used to fill a 7 × 7 × 8 cm plastic pot with drainage holes at the bottom. Seeds from the collected populations were evenly distributed in a bowl, covered with a thin layer of fine soil to ensure no seeds were visible, and watered until the soil was fully saturated. The bowls were then placed in a greenhouse under controlled conditions: 30 ± 5 °C during the day and 20 ± 5 °C at night. When the plants reached the 2–3 leaf stage, the weeds were thinned to eight plants per pot. At the 4-leaf stage, leaves and stems were sprayed with half of the recommended field dose, while control plants were sprayed with water. Fourteen days after treatment, the fresh weight of the above-ground parts of the plants was measured and recorded. Each treatment had four replicates, and the entire experiment was repeated twice.

#### 4.2.2. Whole Plant Dose–Response Assay

The *A. retroflexus* populations, which exhibited suspected resistance and good growth status, were subjected to dose–response curve analysis. For the suspected resistant populations, the concentration gradient was set to 0, 70.31, 140.62, 281.26, 562.52, 1125.04 g a.i./ha; for the sensitive populations, it was set to 0, 1.10, 2.20, 4.39, 8.79, 17.58, 35.16, 70.31, 140.62 g a.i./ha. The bioassay data from whole plant experiments were statistically analyzed. The fresh weight inhibition rate was calculated, and the growth reduction (GR_50_) value was determined using DPS software version 21.05 (Hangzhou Ruifeng Information Technology Co., Ltd., Hangzhou, China). Based on the GR_50_ values obtained, the relative resistance index (RI) was calculated.

Fresh weight inhibition rate (%) = [(control fresh weight − treatment fresh weight)/control fresh weight] × 100%

Relative resistance index = GR_50_ (resistant)/GR_50_ (sensitive)

### 4.3. PPO2 Sequencing

The leaves of individual plants with good growth condition after 21 days of treatment were selected for sampling. The leaf samples were ground into fine powder under liquid nitrogen using a ZHWY-200B thermostatic oscillator. Total RNAs were extracted using the RNA simple Total RNA Kit (Tiangen, DP419). Agarose gel electrophoresis confirmed the integrity of total RNAs. The intact RNA samples were then reverse-transcribed. The first strand of complementary DNA (cDNA) was synthesized using the HiScript^®^ III 1st Strand cDNA Synthesis Kit (+gDNA wiper) (Vazyme, R312-01). The synthesized cDNA from each sample was then stored at −20 °C. The *ppo2* gene of *A. retroflexus* was amplified using the following primers: *ppo2*-F: 5′-GGTACCATGGTAATTCAATCCATTAC-3′ and *ppo2*-R: 5′-TCTAGATTATGCGGTCTTCTCATTC-3′, to amplify the full-length *ppo2* gene (Huang et al., 2020) [40].

PCR was performed in an Applied Biosystems™ Thermo Fisher Scientific thermocycler at a final reaction volume of 50 μL: 5 μL template cDNA, 2 μL each primer, 25 μL 2× T5 Super PCR Mix and 16 μL enzyme-free water. The PCR reaction procedure was as follows: 95 °C for 5 min; 95 °C for 15 s, 58 °C for 15 s, 72 °C for 1 min, 35 cycles; 72 °C for 5 min. PCR amplification products were sequenced by Beijing Tsingke Biotech Co., Ltd., Beijing, China. SnapGene version 6.0.2 was used for sequence analysis.

### 4.4. KASP Assay with RNA as the Template

#### 4.4.1. Design and Synthesis of KASP Primers Using cDNA as Template

Three primers, including two forward primers, F1 comprising F-RNA-HEX and F-RNA-FAM and one reverse primer (R1) designed on the mutant base of *ppo2* gene in *A. retroflexus*, were synthesized for this KASP assay. The following were used: Specific forward primer F-RNA-HEX with HEX fluorescent label: 5′-GAAGGTCGGAGTCAACGGATTTAGAAGCAACAGTTGCCAATTTCACAAAATAAAAG-3′; Specific forward primer F-RNA-FAM with FAM fluorescent label: 5′-GAAGGTGACCAAGTTCATGCTAAGCAACAGTTGCCAATTTCACAAAATAAAGG-3′; Com(R1): 5′-GGTAGTAGCACCGGAAGACCAT-3′; underlined sequences denote specific fluorophore sequences.

#### 4.4.2. Construction of Homozygous Plasmids

We selected a single strain from the resistant population and sequenced it to ensure that it only had Arg-128-Gly mutation. For this, we used pClone007 (Beijing Tsingke Biotech Co., Ltd., Beijing, China), which was connected to DH5α receptive *Escherichia coli* (Vazyme Biotech Co., Ltd., Nanjing, China) to extract Plasmid_R. The extracted plasmid was named Plasmid_R. At the same time, a single strain was selected from the sensitive population and sequenced to ensure that it did not have any mutation, and the plasmid was extracted according to the above method and named Plasmid_S. At the same time, Plasmid_R and Plasmid_S were respectively allocated the same amount of DNA solution to form the artificially constructed heterozygous mutant Plasmid_R+S. For specific steps, see the pClone007 Simple Vector Kit instructions.

#### 4.4.3. Experimental Procedure

Detailed operational procedures of the KASP assay (KASP 2× Master Mix V5 Standard Rox TF from LGC Group) are as follows. Prepare the Primer Mix according to the following steps: primers with a concentration of 100 mM were premixed in the ratio of FAM: HEX: Com = 2:2:5. Subsequently, 54 μL of this premixed solution was combined with 46 μL of distilled water to achieve a final total volume of 100 μL for the Primer Mix. Thaw and gently mix the Master Mix in the kit: briefly centrifuge to concentrate the reagent at the bottom of the tube, then store on ice. Prepare the PCR reaction solution in 96-well plate and configure it on ice: 0.14 μL Primer Mix, 5 μL of Master Mix, and 5 μL of cDNA sample to each well, resulting in a final volume of 10.14 μL per well. PCR reaction was performed according to the following conditions and placed in real-time PCR system for reaction: The PCR conditions were as following: initial denaturation at 94 °C for 15 min; the first amplification step involved denaturation at 94 °C for 20 s, annealed at 65 °C~57 °C and extended for 60 s, a total of 10 cycles; the first cycle was 65 °C, and the temperature of annealing and extension in each subsequent cycle was reduced by 0.8 °C, and dropped to 57 °C after 10 cycles. In the second step, the reaction was denatured at 94 °C for 20 s, annealed at 57 °C, and extended for 60 s for 26 cycles. Fluorescence detection was conducted on the amplified products following the completion of the reaction using a Fluorescent quantitative PCR instrument from Thermo Fisher Scientific, Waltham, MA, USA (Figure 1).

After reading the plate, the fluorescence signal intensity values for FAM, HEX, and ROX in each well were recorded; the data were imported into GraphPad Prism version 10 for processing and visualization as a scatter plot, which facilitated the determination of sample genotypes. FAM values were plotted on the *Y*-axis while HEX values were plotted on the *X*-axis. Normalization was performed using the ROX values from each well in the 96-well plate by dividing the FAM and HEX values by the corresponding ROX values, leading to tighter clustering of data points. cDNA samples sharing the same base at the mutation site will cluster together. Specifically, if only significant FAM fluorescence was detected without significant HEX fluorescence, it indicated that the measured cDNA sample was a homozygous mutant, clustering near the *Y*-axis (FAM axis). Conversely, if only significant HEX fluorescence was detected without significant FAM fluorescence, the measured cDNA sample was a homozygous wild-type, clustering near the *X*-axis (HEX axis). If both significant FAM and HEX fluorescence were detected simultaneously, it suggested that the tested cDNA sample was a heterozygous mutant, clustering in the central region between the axes.

### 4.5. Identification of Consensus DNA Sequence Within PPO2 Genomic Sequence of Different Populations

#### 4.5.1. Preparation of Genomic DNA from *A. retroflexus*

Add two small steel balls into a 2 mL centrifuge tube, followed by fresh leaf tissue. Immediately immerse the tube in liquid nitrogen and freeze the ball mill accessories together for approximately 3 min. Process using the ball mill at 1700 rpm for three cycles of 30 s each to ensure complete homogenization of the plant tissue. After ball milling, add 590 μL of CTAB to the sample and incubate in a water bath at 60 °C for 30 min, mixing every 10 min. Centrifuge at 12,000 rpm for 5 min and transfer the supernatant to a new 1.5 mL centrifuge tube. Add an equal volume of chloroform and isoamyl alcohol (24:1) mixture, then centrifuge at 12,000 rpm for 1 min. Transfer the upper aqueous phase to a fresh 1.5 mL centrifuge tube. Add 0.7 times the volume of cold isopropyl alcohol and incubate at −20 °C for 2 h. Centrifuge at 12,000 rpm for 10 min, after which a white precipitate should be visible at the bottom. Discard the supernatant, wash the pellet with 500 μL of ice-cold 70% ethanol, and centrifuge at 12,000 rpm for 5 min. Carefully discard the supernatant, and place it on a super-clean bench to air dry. Resuspend the DNA in 30–50 μL of sterile water, clean the inner wall, mix thoroughly, and store the sample at −20 °C.

#### 4.5.2. Primer Design and Synthesis

After aligning the DNA sequence (NC_080059.1, positions 8666371-8678257) of a related *Amaranthus* species from NCBI with the cDNA sequence of *A. retroflexus*, two primers were designed based on highly conserved regions flanking the mutation site: Ar/ppo/partialDNA/F: 5′-ACAGAAAGTGAGGCAGAGGTCTC-3′; Ar/ppo/partialDNA/R: 5′-GATTTTGCTGAAAGGATATTGCTCGTGA-3′.

#### 4.5.3. PPO2 Gene Fragment Amplification and Sequencing

PCR was conducted using an Applied Biosystems™ Thermo Fisher Scientific thermocycler with a final reaction volume of 50 μL, comprising 2 μL template DNA, 2.5 μL of each primer, 25 μL of Vazyme 2× Rapid Taq Master Mix, and 18 μL nuclease-free water. The PCR cycling conditions were as follows: initial denaturation at 95 °C for 5 min; followed by 35 cycles of denaturation at 95 °C for 30 s, annealing at 60 °C for 30 s, and extension at 72 °C for 45 s; with a final extension at 72 °C for 5 min. The amplified products were subsequently sent for DNA sequencing. SnapGene version 6.0.2 was used for sequence analysis.

### 4.6. KASP Assay with Genomic DNA as the Template

#### 4.6.1. Design and Synthesis of KASP Primers Using DNA as Template

Three primers, including two forward primers F2 comprising F-DNA-HEX and F-DNA-FAM and one reverse primer R2, were synthesized based on the consensus sequences of PPO2 around 128 sites. The following were used: Specific forward primer F-DNA-HEX with HEX fluorescent label: 5′-GAAGGTCGGAGTCAACGGATTTATCTTCTGTCACAGCCAATTTCACAAAATAAAAG-3′; Specific forward primer F-DNA-FAM with FAM fluorescent label: 5′-GAAGGTGACCAAGTTCATGCTTCTTCTGTCACAGCCAATTTCACAAAATAAAGG-3′; R2: 5′-ACTTACTAGCACCGGAAGACCAT-3′; underlined sequences denote specific fluorophore sequences.

#### 4.6.2. Experimental Procedure

The operations are the same as those in Section 4.4.3, except for different templates.

## Data Availability

The data that support the findings of this study are available upon reasonable request from the corresponding author.

## Supplementary Materials

The following supporting information can be downloaded at: https://www.mdpi.com/article/10.3390/plants14040515/s1, Table S1 Resistance detection of *Amaranthus retroflexus* populations.

## References

[1] Li W. (2015). Studies on Damage Investigation and Chemical Control of Soybean Weeds in the North in the North of Heilongjiang Province.

[2] Ding Y., Sun H. (2024). Operation situation of domestic and foreign soybean market in 2023 and Outlook in 2024. China Grain Econ..

[3] Zhao Y., Zhang Q., Zheng P., Tian Q., Wu Y. (1995). Investigation and chemical control of weeds in soybean field. Chin. Plant Prot..

[4] Andini R., Sulaiman M.I., Ohsawa R. (2018). Natural polyploidy in amaranths (*Amaranthus* spp.). AIP Conf. Proc..

[5] Xiang G., Wang Y., Peng Y. (2010). Investigation on species, distribution and harm of exotic Amaranth plants in Dongting Lake District. Guizhou Nongye Kexue.

[6] Costea M., Weaver S.E., Tardif F.J. (2004). The biology of Canadian weeds. 130. *Amaranthus retroflexus* L., *A. powellii*, *S. Watson* and *A. hybridus* L.. Can. J. Plant Sci..

[7] Su S. (2011). Herbicide use and change in soybean field. Mod. Agrochem..

[8] Wang X. (2019). Study on Resistance of *Amaranthus retroflexus* to Flursulfamethoxane in Soybean Field.

[9] Hao G.F., Zuo Y., Yang S.G., Yang G.F. (2011). Protoporphyrinogen oxidase inhibitor: An ideal target for herbicide discovery. Chimia.

[10] Arnould S., Takahashi M., Camadro J.M. (1999). Acylation stabilizes a protease-resistant conformation of protoporphyrinogen oxidase, the molecular target of diphenyl ether-type herbicides. Proc. Natl. Acad. Sci. USA.

[11] Lermontova I., Kruse E., Mock H.P., Grimm B. (1997). Cloning and characterization of a plastidal and a mitochondrial isoform of tobacco protoporphyrinogen IX oxidase. Proc. Natl. Acad. Sci. USA.

[12] Duke S.O., Becerril J.M., Sherman T.D., Lydon J., Matsumoto H. (1990). The role of protoporphyrin IX in the mechanism of action of diphenyl ether herbicides. Pestic. Sci..

[13] Guo J., Lu Y., Sun J. (2000). Residual dynamics of flursulfamethofen in peanut and soybean fields. Agric. Environ. Prot..

[14] Zhong J. (2005). Production and field application of diphenyl ether herbicides. Agrochemical.

[15] Powles Stephen B., Yu Q. (2010). Evolution in action: Plants resistant to herbicides. Annu. Rev. Plant Biol..

[16] Heap I. The International Herbicide-Resistant Weed Database. https://weedscience.org/Pages/filter.aspx.

[17] Shoup D.E., Al-Khatib K., Peterson D.E. (2003). Common waterhemp (*Amaranthus rudis*) resistance to protoporphyrinogen oxidase-inhibiting herbicides. Weed Sci..

[18] He F., Dai L., Qu C. (2011). Screening of foliar-applied herbicides for controlling Acalypha australis in soybean fields. Plant Prot..

[19] Teng C., Cui S., Tan H., Zhang J., Cao B., Li Q., Tao B. (2016). Resistance of *Amaranthus retroflexus* to fomesafen in soybean fields of Heilongjiang Province. Agrochemical.

[20] Patzoldt W.L., Hager A.G., McCormick J.S., Tranel P.J. (2006). A codon deletion confers resistance to herbicides inhibiting protoporphyrinogen oxidase. Proc. Natl. Acad. Sci. USA.

[21] Giacomini D.A., Umphres A.M., Nie H., Mueller T.C., Steckel L.E., Young B.G., Scott R.C., Tranel P.J. (2017). Two new PPX2 mutations associated with resistance to PPO-inhibiting herbicides in *Amaranthus palmeri*. Pest Manag. Sci..

[22] Wang H. (2020). Resistance Mechanism of *Amaranthus retroflexus* L. to Fomesafen and Nicosulfuron in Corn-Soybean Rotation Area.

[23] Cao Y., Huang H., Wei S., Lan Y., Li W., Sun Y., Wang R., Huang Z. (2022). Target gene mutation and enhanced metabolism confer fomesafen resistance in an *Amaranthus retroflexus* L. population from China. Pestic. Biochem. Physiol..

[24] Wu Q., Wei J., Guo J., Zhang Z., Feng Z., Chen J. (2024). Resistance level and molecular mechanism of *Amaranthus retroflexus* L. to fomesafen. J. Nanjing Agric. Univ..

[25] Rangani G., Salas-Perez R.A., Aponte R.A., Knapp M., Craig I.R., Mietzner T., Langaro A.C., Noguera M.M., Porri A., Roma-Burgos N. (2019). A Novel Single-Site Mutation in the Catalytic Domain of Protoporphyrinogen Oxidase IX (PPO) Confers Resistance to PPO-Inhibiting Herbicides. Front. Plant Sci..

[26] Nie H., Harre N.T.T., Young B.G.G. (2023). A New V361A Mutation in *Amaranthus palmeri PPX2* Associated with PPO-Inhibiting Herbicide Resistance. Plants.

[27] Bi B., Wang Q., Coleman J.J., Porri A., Peppers J.M., Patel J.D., Betz M., Lerchl J., McElroy J.S. (2020). A novel mutation A212T in chloroplast Protoporphyrinogen oxidase (PPO1) confers resistance to PPO inhibitor Oxadiazon in Eleusine indica. Pest Manag. Sci..

[28] Fu Z., Zhang C., Qian Y., Hu X., Zhang J. (1999). Several detection methods of resistant weeds. Plant Prot..

[29] Wang Q., Dong L., Lou Y., Zhang S. (2002). Detection and identification methods of weed resistance in farmland. J. Weed Sci..

[30] Patterson E.L., Fleming M.B., Kessler K.C., Nissen S.J., Gaines T.A. (2017). A KASP Genotyping Method to Identify Northern Watermilfoil, Eurasian Watermilfoil, and Their Interspecific Hybrids. Front. Plants Sci..

[31] Broccanello C., Chiodi C., Funk A., McGrath J.M., Panella L., Stevanato P. (2018). Comparison of three PCR-based assays for SNP genotyping in plants. Plant Methods.

[32] Rosas J.E., Bonnecarrere V., Perez De Vida F. (2014). One-step, codominant detection of imidazolinone resistance mutations in weedy rice (*Oryza sativa* L.). Electron. J. Biotechnol..

[33] Sarangi D., Stephens T., Barker A.L., Patterson E.L., Gaines T.A., Jhala A.J. (2019). Protoporphyrinogen oxidase (PPO) inhibitor–resistant waterhemp (*Amaranthus tuberculatus*) from Nebraska is multiple herbicide resistant: Confirmation, mechanism of resistance, and management. Weed Sci..

[34] Ashigh J., Corbett C.A.L., Smith P.J., Laplante J., Tardif F.J. (2009). Characterization and diagnostic tests of resistance to acetohydroxyacid synthase inhibitors due to an Asp376Glu substitution in *Amaranthus powellii*. Pestic. Biochem. Physiol..

[35] Rousonelos S.L., Lee R.M., Moreira M.S., VanGessel M.J., Tranel P.J. (2012). Characterization of a Common Ragweed (*Ambrosia artemisiifolia*) Population Resistant to ALS- and PPO-Inhibiting Herbicides. Weed Sci..

[36] Steckel L.E. (2007). The Dioecious Amaranthus spp.: Here to Stay. Weed Technol..

[37] Cao S. (2024). Study on the Resistance Mechanism of Ipomoea Nil to Fomesafen.

[38] Du L., Li N., Shang S., Li D., Li X., Qu M. (2024). Study on mechanism of multiple resistance and target resistance to herbicides in soybean field. Plant Prot..

[39] Wang H., Wang H., Zhao N., Zhu B., Sun P., Liu W., Wang J. (2019). Multiple Resistance to PPO and ALS Inhibitors in Redroot Pigweed (*Amaranthus retroflexus*). Weed Sci..

[40] Huang Z., Cui H., Wang C., Wu T., Zhang C., Huang H., Wei S. (2020). Investigation of resistance mechanism to fomesafen in *Amaranthus retroflexus* L.. Pestic. Biochem. Physiol..

[41] Varanasi V.K., Brabham C., Norsworthy J.K. (2018). Confirmation and Characterization of Non–target site Resistance to Fomesafen in Palmer amaranth (*Amaranthus palmeri*). Weed Sci..

[42] Obenland O.A., Ma R., O’Brien S.R., Lygin A.V., Riechers D.E. (2019). Carfentrazone-ethyl resistance in an Amaranthus tuberculatus population is not mediated by amino acid alterations in the PPO2 protein. PLoS ONE.

[43] Kaundun S.S., Hutchings S.J., Dale R.P., Bailly G.C., Glanfield P. (2011). Syngenta ‘RISQ’ test: A novel in-season method for detecting resistance to post-emergence ACCase and ALS inhibitor herbicides in grass weeds. Weed Res..

