# Supplementary material for: Rapid Resistance Detection of Amaranthus retroflexus to Fomesafen via Kompetitive Allele-Specific PCR (KASP)
Source: Plants (Basel). 2025 Feb 8;14(4):515. doi: 10.3390/plants14040515 (PMC11858902; doi:10.3390/plants14040515)
Supplement: Supplementary file 1 [file plants-14-00515-s001.zip › plants-3427775-supplementary.pdf]

Table S1 Resistance detection of *Amaranthus retroflexus* populations

Population number	Collection year	Collection site			Germination rate	Whole plant dose-response assay			DNA Sequencing		KASP
		Province	City	County / Town / Village		Fresh weight inhibition rate	GR_{50}	RI	Mutation	Mutation frequency	Fluorescence signal
FZ-1	2014	Jiangsu	Nanjing	Jiangpu farm	5%	100%	/	/	No mutation	0/10	HEX
FZ-2	2014	Henan	Xuchang	Laocheng town	4%	100%	/	/	No mutation	0/10	HEX
FZ-3	2017	Heilongjiang	Heihe	Nenjiang first administration area	16%	37.50%	/	/	AGG-128-GGG	10/10	FAM
FZ-4	2017	Heilongjiang	Heihe	Nenjiang first administration area	20%	75%	/	/	AGG-128-GGG	10/10	FAM
FZ-5	2017	Heilongjiang	Heihe	Nenjiang second administration area	52%	6.25%	115.38	40.72	AGG-128-GGG	10/10	FAM
FZ-6	2017	Heilongjiang	Heihe	Nenjiang third administration area	12%	27.68%	/	/	AGG-128-GGG	10/10	FAM
FZ-7	2017	Heilongjiang	Heihe	Nenjiang fourth administration area	76%	18.75%	115.80	40.87	AGG-128-GGG	10/10	FAM
FZ-8	2017	Heilongjiang	Heihe	Nenjiang fourth administration area	68%	28.13%	110.41	38.97	AGG-128-GGG	10/10	FAM
FZ-9	2017	Heilongjiang	Heihe	Nenjiang fifth administration area	20%	53.13%	/	/	AGG-128-GGG	10/10	FAM
FZ-10	2017	Heilongjiang	Heihe	Nenjiang fifth administration area	20%	40.63%	/	/	AGG-128-GGG	10/10	FAM
FZ-11	2017	Heilongjiang	Heihe	Nenjiang sixth administration area	54%	40.63%	125.56	44.31	AGG-128-GGG	10/10	FAM
FZ-12	2017	Heilongjiang	Heihe	Nenjiang seventh administration area	8%	37.68%	/	/	AGG-128-GGG	10/10	FAM
FZ-13	2017	Heilongjiang	Heihe	Nenjiang seventh administration area	12%	51.25%	/	/	AGG-128-GGG	10/10	FAM
FZ-14	2017	Heilongjiang	Heihe	Nenjiang eighth administration area	22%	53.13%	/	/	AGG-128-GGG	10/10	FAM
FZ-15	2017	Heilongjiang	Heihe	Nenjiang eighth administration area	20%	52.75%	/	/	AGG-128-GGG	10/10	FAM
FZ-16	2018	Jiangsu	Lianyungang	Ganyv western of maban road	48%	96.88%	9.73	3.43	No mutation	0/10	HEX
FZ-17	2018	Jiangsu	Yangzhou	Jiangdu district xiannv town	44%	100%	/	/	No mutation	0/10	HEX
FZ-18	2019	Jiangsu	Yangzhou	Jiangdu dinghuo town	46%	100%	16.40	5.79	No mutation	0/10	HEX
FZ-19	2020	Jiangsu	Suqian	Yanghe winery	82%	100%	/	/	No mutation	0/10	HEX
FZ-20	2020	Jiangsu	Nanjing	Lishui district baima farm	8%	100%	/	/	No mutation	0/10	HEX
FZ-21	2020	Jiangsu	Huaian	Hongze fangte road	100%	100%	/	/	No mutation	0/10	HEX
FZ-22	2020	Jiangsu	Suqian	Qianzhang village	56%	100%	20.28	7.16	No mutation	0/10	HEX
FZ-23	2020	Jiangsu	Yangzhou	Gaoyou county liuan village	74%	93.75%	/	/	No mutation	0/10	HEX
FZ-24	2021	Anhui	Fuyang	Jieshou town niema village	90%	87.50%	/	/	No mutation	0/10	HEX
FZ-25	2020	Anhui	Wuhu	Wuwei county nicha town	72%	100%	19.11	6.75	No mutation	0/10	HEX
FZ-26	2022	Jiangsu	Yangzhou	Jiangdu town tuanjie village	90%	100%	/	/	No mutation	0/10	HEX
FZ-27	2022	Jiangsu	Yangzhou	Jiangdu town tuanjie village	87%	100%	/	/	No mutation	0/10	HEX
FZ-28	2023	Anhui	Suzhou	Yongqiao county zhihe town jieqiao village	100%	80%	/	/	No mutation	0/10	HEX
FZ-29	2023	Anhui	Suzhou	Yongqiao county gaoxiangzi village	90%	79%	/	/	No mutation	0/10	HEX
FZ-30	2023	Anhui	Suzhou	Yongqiao county 050 Township Road	90%	80%	10.99	3.88	No mutation	0/10	HEX
FZ-31	2023	Jiangsu	Nanjing	Lishui district baima farm	80%	100%	/	/	No mutation	0/10	HEX
FZ-32	2023	Jiangsu	Xuzhou	Suining county dazhao village	90%	70%	/	/	No mutation	0/10	HEX
FZ-33	2023	Jiangsu	Xuzhou	Suining county gaozhao village	100%	90%	15.92	5.62	No mutation	0/10	HEX
FZ-34	2023	Jiangsu	Xuzhou	Suining county houlou village	100%	100%	/	/	No mutation	0/10	HEX
FZ-35	2023	Jiangsu	Xuzhou	Suining county geduan village	100%	100%	/	/	No mutation	0/10	HEX
FZ-36	2023	Heilongjiang	Jiamusi	Fujin jianghe farm first administration area	90%	100%	2.98	1.05	No mutation	0/10	HEX
FZ-37	2023	Henan	Shangqiu	Xiayi county qihe village	100%	97%	10.15	3.58	No mutation	0/10	HEX
FZ-38	2023	Shandong	Jining'	Wensahng county yuanzhuang town	70%	94%	/	/	No mutation	0/10	HEX
FZ-39	2023	Heilongjiang	Haerbin	Bin town	70%	100%	3.98	1.4	No mutation	0/10	HEX
FZ-40	2023	Anhui	Fuyang	Yingshang county chuigang village	100%	95%	/	/	No mutation	0/10	HEX
FZ-41	2023	Heilongjiang	Mishan	Mudanjiang Administration 856 farm	100%	100%	2.83	1	No mutation	0/10	HEX
FZ-42	2023	Henan	Shangqiu	Yoncheng county dawangji town	100%	100%	11.22	3.96	No mutation	0/10	HEX
